# Preparation of Poly Aluminum-Ferric Chloride (PAFC) Coagulant by Extracting Aluminum and Iron Ions from High Iron Content Coal Gangue

**DOI:** 10.3390/ma15062253

**Published:** 2022-03-18

**Authors:** Deshun Kong, Zihan Zhou, Shuojiang Song, Shan Feng, Minglei Lian, Rongli Jiang

**Affiliations:** 1 Guizhou Provincial Key Laboratory of Coal Clean Utilization, School of Chemistry and Materials Engineering, Liupanshui Normal University, Liupanshui 553004, China; lb19040009@cumt.edu.cn (D.K.); lb20040020@cumt.edu.cn (S.S.); 20132013071@cqu.edu.cn (S.F.); feiyuhu2003@126.com (M.L.); 2 School of Chemical Engineering and Technology, China University of Mining and Technology, Xuzhou 221016, China; tb19040017b2@cumt.edu.cn

**Keywords:** high iron content coal gangue, acid leaching, extraction of aluminum and iron ions, PAFC

## Abstract

Poly aluminum-ferric Chloride (PAFC) is a new type of high efficiency coagulant. In this study, high iron type gangue is used as a main raw material. It is calcined at 675 °C for 1 h and 3% CaF_2_ is added to the calcined powder and reacted with 20% hydrochloric acid at 93 °C for 4 h. The leaching ratio of aluminum ions is 90% and that of iron ions is 91%. After Fe^2+^ ions are oxidized in the filtrate, CaCO_3_ is used to adjust the pH of the filtrate to 0.7. The microwave power is adjusted to 80 W and the filtrate is radiated for 5 min. After being aged for 24 h, PAFC product is obtained. The prepared PAFC is used to treat mine water and compared with the results of PAC and PAF, the turbidity removal ratio of PAFC is 99.6%, which is greater than 96.4% of PAC and 93.7% of PAF. PAFC is a mixture with different degrees of polymerization. It demonstrates that extracting aluminum and iron ions from high iron content gangue to prepare PAFC by microwave is efficient and feasible.

## 1. Introduction

The coal gangue accounts for about 10–20% of coal production, and it is one of the largest industrial solid wastes emitted in China [1,2]. It occupies land and pollutes the atmosphere and water [3,4], so carrying out comprehensive utilization of coal gangue is one of the important ways to solve these problems. The traditional utilization methods are mainly focused on power generation [5], construction materials production [6,7], mine filling [8,9], road construction, and land reclamation [10]; these utilization methods consume a relatively large amount of coal gangue, but there are problems such as lower technology and lower added value and easy to produce secondary pollution.

Coal gangue is a waste resource containing aluminum, iron, and silicon elements, which contain kaolinite, quartz, hematite, rutile, and so on [11], converted into oxides mainly including SiO_2_, Al_2_O_3_, Fe_2_O_3_, CaO, MgO, TiO_2_, etc. The components vary according to location due to different geological conditions. SiO_2_ and Al_2_O_3_ are their main components, and their total amounts (mass fraction) can reach 60–90% [12]. Therefore, coal gangue provides great potential in resource utilization, extracting aluminum and iron elements to prepare new products is an important research direction [13].

Liupanshui City (Guizhou Province, China) produces over 10 million tons of gangue each year; this amount has exceeded 200 million tons when the accumulation of previous years is taken into account. Compared to other regions in China, coal gangue from Liupanshui has high iron content and medium aluminum content and is reddish after calcination, making it unsuitable for making products requiring high whiteness, such as ceramics [14]; this further narrows the scope for utilization. However, coal gangue is a rejected resource of aluminum and iron, from which aluminum and iron ions can be extracted to expand the applications of gangue. It makes up for the lack of resources such as bauxite and iron ore.

At the same time, the coal industry produces a large amount of wastewater that needs to be treated. The particles suspended in this wastewater are often negatively charged, and aluminum and iron salts are often used as coagulants due to their remarkable electrical neutralization properties. In recent years, inorganic high molecular weight coagulants have garnered significant interest as water purifying agents [15], especially PAC [16]. Poly aluminum-ferric chloride (PAFC) is a highly efficient inorganic coagulant, which is a polymer formed by Al^3+^ and Fe^3+^ with high molecular weight, high charge and strong bridging effect [17], and has the advantages of both aluminum and iron salts [18,19], so it is more effective than traditional inorganic coagulants [20,21], and it has a wider pH range for use than single aluminum and iron salts [22]. Thus, it has been widely used [23,24,25,26,27].

PAFC is mostly prepared from FeCl_3_ and AlCl_3_, which is more costly, and the mineral synthesis of PAFC and other coagulants have also been reported [28,29,30,31], but they are mostly polymerized by hydrothermal methods for several hours, which are less efficient.

In this work, aluminum and iron ions are extracted from gangue calcined powder by acid leaching with hydrochloric acid, and the extraction ratio of both aluminum and iron elements in it exceeded 90%, solving the problem that aluminum ions are difficult dissolve. PAFC is prepared by microwave synthesis with a shortened time of 5 min and the conditions of preparation are optimized. Its performance is better than that of the commonly used polymeric aluminum chloride (PAC) and polymeric iron chloride (PAF).

## 2. Materials and Methods

### 2.1. Materials

The gangue sample comes from a coal mine in Liupanshui City. Thirty percent H_2_O_2_, CaF_2_, and CaCO_3_ are the analytical purity reagents from Tianjin City Zhiyuan Chemical Co., Ltd. Tianjin, China. Hydrochloric acid is an analytical purity reagent from Chongqing Chuandong Chemical Co., Ltd. Chongqing, China. Polymeric aluminum chloride (PAC), polymeric iron chloride (PAF), and kaolinite are bought from the market, they are chemically pure reagents.

### 2.2. Procedure

The gangue is crushed and ground, then passed through a 160 mesh sieve, followed by calcination at 675 °C for 1 h. After taking out the calcined powder and cooling it, hydrochloric acid with a mass fraction of 20% is added following the solid–liquid ratio of 1:4.5, and then calcium fluoride powder is added at 3% of the mass of the gangue, and the reaction is carried out at 93 °C for 4 h under stirring conditions, and the acid leach solution is obtained by filtration.

**Figure 1:**
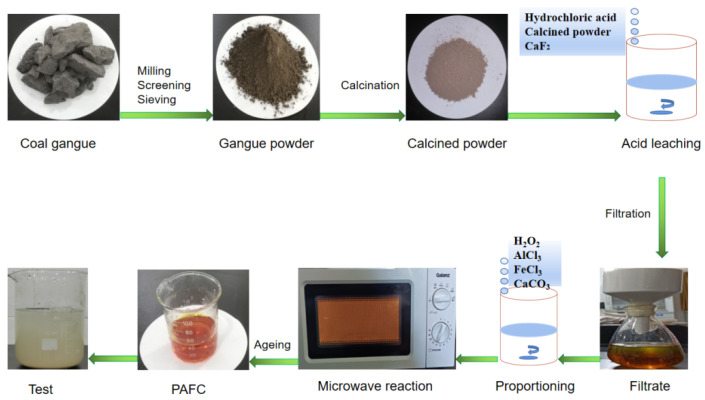
Flow chart of the experiments.

After adding hydrogen peroxide to the filtrate to oxidize Fe^2+^ to Fe^3+^, calcium carbonate powder is used to adjust the pH of the acid leach solution. An amount of 100 mL of the above solution is taken and placed in a microwave oven for 2–7 min to polymerize, and is then aged for 24 h to obtain a polymerized PAFC solution, which is then tested for its turbidity removal properties. The process flow diagram for the preparation is shown in Figure 1.

### 2.3. Instrumentation and Characterization

A 6100 type X-ray diffraction instrument (XRD, Shimadzu Company, Kyoto, Japan) is used for CuKα (λ for Kα = 1.54059 Å), 2θ = 3°–65°, with a step width of 0.02°. The main components are determined by a Supermini200 type X-ray fluorescence spectrometer (XRF, Rigaku Company, Tokyo, Japan). The morphology of the materials is identified by a Zeiss EVO18-type scanning electron microscope (SEM, Jena, Germany). 7600 type fourier transform infrared spectroscopy (FT-IR, Tianjin Gangdong Sci&Tech Company, Tianjin, China). WGZ-1S type desktop turbidimeter (Shanghai Xinrui Instrument Company, Shanghai, China). P70J17l-V1 type microwave oven (Galanz Company, Guangzhou, China).

### 2.4. Source of Wastewater

(1) Simulated wastewater: 3.0000 g of 5000 mesh kaolin powder is added to a 1000 mL beaker and filled with 800 mL of distilled water, stirred well and then the pH is adjusted to 7.0 with sodium hydroxide solution to obtain simulated wastewater with an initial turbidity of 3750 NTU.

(2) Mine wastewater: from a coal mine in Liupanshui City, with an initial turbidity of 4896 NTU.

### 2.5. Determination of Turbidity Removal Performance

An amount of 5 mL of PAFC solution with a mass fraction of 1% is added to 800 mL of simulated wastewater, first stirred at a rate of 1000 r/min for 3 min, then at a rate of 200 r/min for 3 min, and after standing for 30 min, the supernatant is taken to measure the turbidity, then the turbidity removal ratio of the wastewater is calculated. Its calculation formula is as follows:

Ratio = [(T_1_ − T_2_)/T_1_] × 100%

T_1_: initial turbidity, T_2_: residual turbidity.

## 3. Results and Discussion

### 3.1. XRD Analysis of Coal Gangue

**Figure 2:**
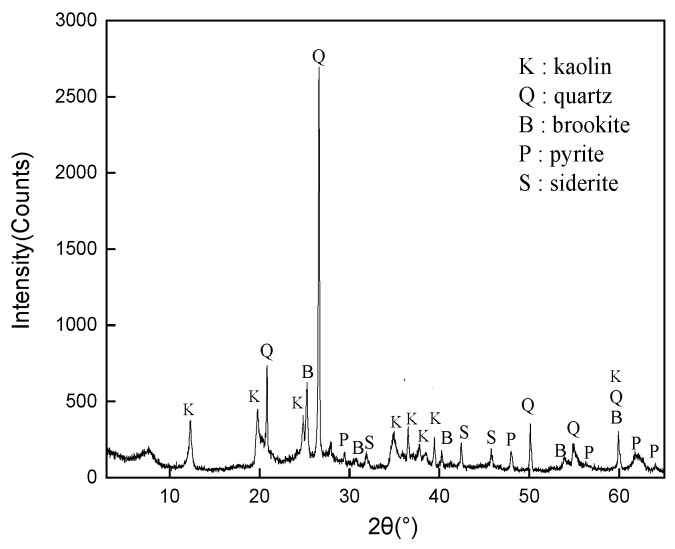
XRD spectrum of coal gangue.

As can be seen from Figure 2, the main crystalline materials in this gangue sample are kaolinite, quartz, plagioclase, pyrite, and siderite, etc. It can be seen that the component of this sample is complex and the main elements that make up these minerals are aluminum, iron, silicon, and titanium.

### 3.2. Morphology and Energy Spectrum Analyses of the Coal Gangue

**Figure 3:**
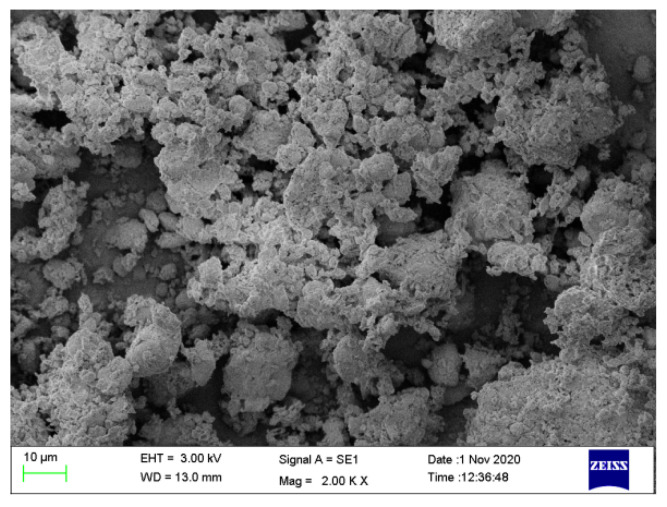
SEM image of the gangue [13].

**Figure 4:**
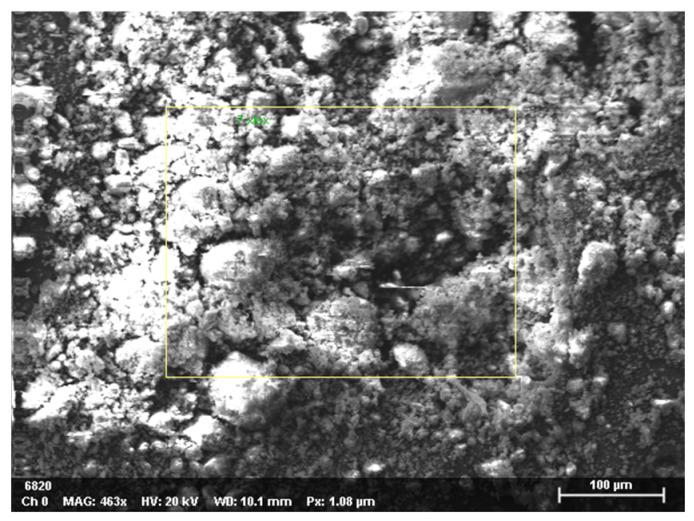
XPS scanning range of coal gangue.

**Figure 5:**
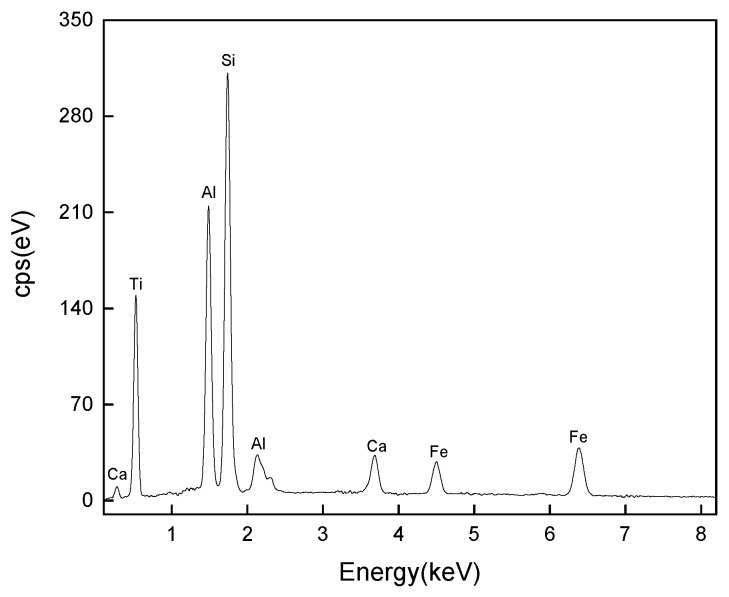
XPS diagram of the coal gangue.

From Figure 3, it can be seen that the crushed raw coal gangue particles have an irregular shape, the smallest particle diameter is about 1 micron, the larger particles are about tens of microns, and there is an agglomeration phenomenon; the smaller particles are conducive to the acid leaching reaction. To understand the elements inside the mineral, a region within the yellow box as shown in Figure 4 is selected for energy spectrum analysis; the results are shown in Figure 5.

From Figure 5, it can be seen that the main elements in this sample are silicon, aluminum, iron, titanium, and calcium, which is in agreement with the analysis of the XRD spectrum.

### 3.3. XRD Analysis of Calcined Powder and Acid Leaching Residue of Coal Gangue

**Figure 6:**
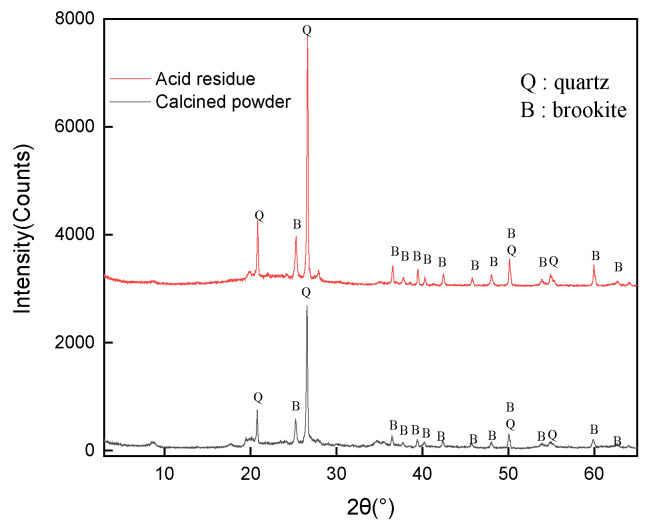
XRD spectra of gangue calcined powder and acid leaching filter residue.

To master the physical phase changes after calcination and acid leaching, XRD analysis of calcined powder and acid leaching filter residue is carried out, and the results are shown in Figure 6.

After calcination, the diffraction peaks of kaolinite, pyrite and siderite disappeared. Kaolinite (Al_2_O_3_·2SiO_2_·2H_2_O) is a silicate with a 1:1 type structure consisting of [SiO_4_] tetrahedral layers joined with [AlO_2_(OH)_4_] octahedral layers [32]. The substances of this crystal structure are chemically inactive and must be activated [33]. Activation methods include mechanical activation, thermal activation [34], microwave irradiation activation [35], and composite activation, among which, high-temperature calcination is one of the most common activation methods [36,37] and is suitable for industrial production.

During high temperature calcination, the crystalline structure of kaolinite is destroyed [38] and the following reactions occur:

Al_2_Si_2_O_5_(OH)_4_ (kaolinite) → Al_2_O_3_·2SiO_2_ (metakaolin) + 2H_2_O ↑

After calcination, the hydrogen and oxygen bonds between the [SiO_4_] tetrahedral layer and the [AlO_2_(OH)_4_] octahedral layer in kaolinite are broken, and the lamellar structure is distorted by disruption [39,40], and kaolinite becomes amorphous metakaolin, which includes amorphous alumina and silica with high activity [41].

Iron-bearing substances such as pyrite undergo the following chemical reaction [42]:

4FeS_2_ (pyrite) + 11O_2_ → 2Fe_2_O_3_ + 8SO_2_

As can be seen from Table 1, the iron content of the gangue is high, but it is difficult to find the diffraction peaks of Fe_2_O_3_ in Figure 6, which indicates that the newly generated Fe_2_O_3_ is mainly amorphous with high chemical activity, which is also conducive to the dissolution of iron ions.

There are various processes for extracting Al_2_O_3_ from gangue [43], more commonly acid and alkali methods are used. Calcined powders are chemically active and Al_2_O_3_ can be extracted by acid [44,45,46,47], with the following reactions occurring.

Al_2_O_3_ + 6H^+^ → 2Al^3+^ + 3H_2_O

Fe_2_O_3_ + 6H^+^ → 2Fe^3+^ + 3H_2_O

The diffraction peak shapes of the filter residue after acid leaching are similar to those before acid leaching, but their intensities are different because quartz and brookite cannot react with dilute hydrochloric acid, and after acid leaching, the dissolution of the aluminum and iron ions causes the relative content of the remaining material to rise, so their diffraction intensities are enhanced.

The added CaF_2_ reacts first with the hydrochloric acid and then with the silica in the following equation.

CaF_2_ + 2HCl → CaCl_2_ + 2HF

SiO_2_ + 6HF → H_2_SiF_6_ + 2H_2_O

The silica ions in the partial kaolinite are dissolved, and the pore channels formed promoted an increase in the dissolution ratio of aluminum ions. Comparative experiments show that the dissolution ratio of aluminum ions without CaF_2_ is up to 61%, and after adding it is 90%, which shows a more obvious effect.

### 3.4. XRD and Composition Analysis of Calcined Powder and Acid Leaching Filter Residue of Coal Gangue

To grasp the content of the elements in this sample, the raw coal gangue, calcined powder, and acid leaching filter residue are analyzed for their composition. The results are shown in Table 1.

As can be seen from Table 1, the content of aluminum and iron elements in the gangue and calcined powder is high, when acid leaching, aluminum and iron ions react with dilute hydrochloric acid, so the content of aluminum and iron becomes low, and it can be calculated that the leaching ratio of aluminum and iron elements are more than 90%, which achieves the extraction of aluminum and iron and other metal elements in the gangue. Titanium and quartz do not react with dilute hydrochloric acid, so the titanium and silicon elements in the filtrate are enriched, resulting in their relative content in the filtrate increasing, which is consistent with the conclusion of Figure 6. The filtrate contained mainly aluminum and iron ions, with a leaching ratio of 90% for aluminum ions and 91% for iron ions. The concentration of aluminum ions in the filtrate is 0.858 mol/L and 0.366 mol/L for iron ions, which could be used to prepare PAFC.

### 3.5. Effect of pH of the Preparation System on the Turbidity Removal Performance

The microwave oven is at 80 W and the pH is adjusted to 0.5, 0.6, 0.7, 0.8, and 0.9, the reactions are carried out in the microwave oven for 5 min and the products are used to treat the simulated wastewater after preparation.

**Figure 7:**
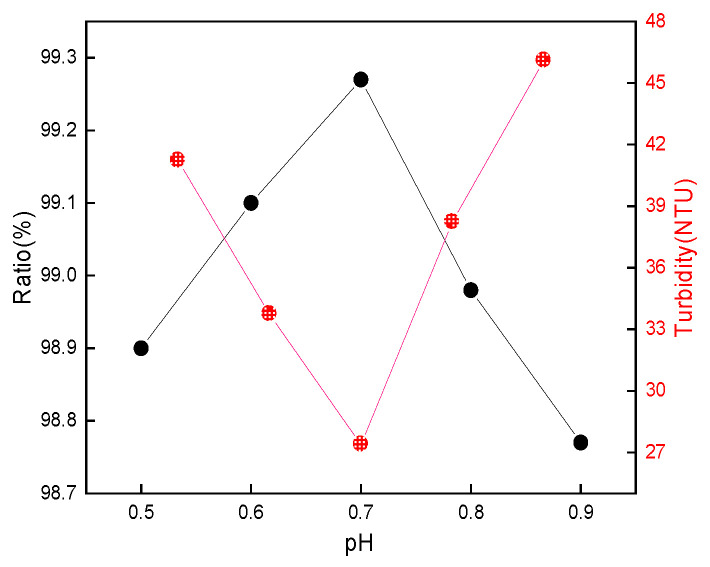
Effect of pH on the turbidity removal ratio.

Figure 7 shows that with the increase of pH, the turbidity removal ratio first increases and then decreases. The maximum turbidity removal ratio is 99.26% at pH = 0.7. Because the pH will affect the degree of hydrolysis of aluminum and iron ions, appropriate pH can promote hydrolysis, generate various hydroxyl aluminum ions and hydroxyl iron ions, and promote the polymerization reaction, the main reactions of the process are as follows [48]:

Al_2_O_3_ + 6HCl → 2AlCl_3_ + 3H_2_O

2AlCl_3_ + nH_2_O → Al_2_(OH)_n_Cl_6−n_ + nHCl

m[Al_2_(OH)_n_Cl_6−n_] → [Al_2_(OH)_n_Cl_6−n_]_m_ (m ≤ 10, n ≤ 5)

Fe_2_O_3_ + 6HCl → 2FeCl_3_ + 3H_2_O

2FeCl_3_ + nH_2_O → Fe_2_(OH)_n_Cl_6−n_ + nHCl

m[Fe_2_(OH)_n_Cl_6−n_] → [Fe_2_(OH)_n_Cl_6−n_]_m_ (m ≤ 10, n ≤ 5)

m_1_[Al_2_(OH)_n1_Cl_6−n_] + m_2_[Fe_2_(OH)_n2_Cl_6−n_] → [Al_2_(OH)_n1_Cl_6−n_]_m1_[Fe_2_(OH)_n2_Cl_6−n_]_m2_ (m_1,2_ ≤ 10, n_1,2_ ≤ 5)

The chemical formula of PAFC shows that the substance is a multimer with aluminum and iron ions as the central ions and hydroxide and chloride ions as the ligands, and due to the different values of m and n, it is a complex mixture of components.

If the pH of the system is too high, the aluminum and iron ions will form mononuclear hydroxyl complexes, which will eventually produce aluminum hydroxide and iron hydroxide precipitates and affect its turbidity removal performance. If the pH is too low, the acidity of the system is high and the hydrolysis of aluminum and iron ions is inhibited, making it difficult to form polymers; if the pH is high, the system can promote the hydrolysis of aluminum and iron ions, but too high a pH will easily produce Fe(OH)_3_ and Al(OH)_3_ precipitates in the system, so pH = 0.7 is chosen.

### 3.6. Effect of Microwave Power on Turbidity Removal Performance

**Figure 8:**
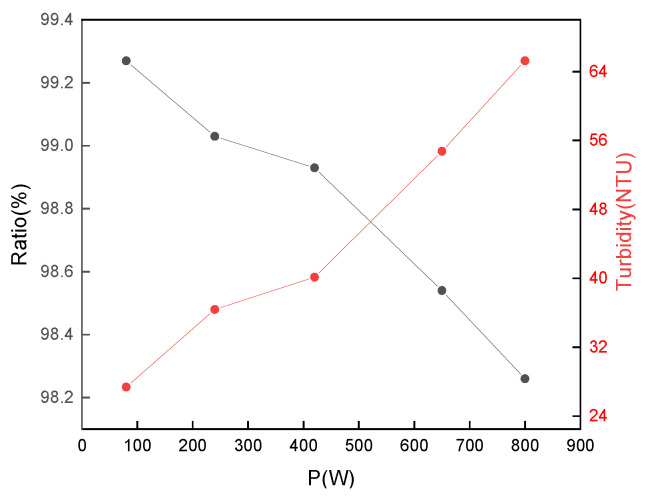
Effect of microwave oven power on the turbidity removal ratio.

The pH is adjusted to 0.7, and the microwave power is changed to 80 W, 240 W, 420 W, 650 W, 800 W, and the reaction is carried out for 5 min, and the product is used to treat the simulated wastewater after preparation, and the results are shown in Figure 8.

As can be seen from Figure 8, the turbidity removal ratio with the increase in power and a decreasing trend, microwave power is 80 W suitable for the highest turbidity removal ratio. As the power determines the polymerization temperature of the product, the power is larger and the temperature of the reaction system is higher [49], which will lead to the system of aluminum and iron ions in violent hydrolysis, thus producing aluminum hydroxide, iron hydroxide, and other precipitation;, aluminum and iron ions cannot be well polymerized, so the product turbidity removal performance is poor and the choice power is 80 W.

### 3.7. Effect of Radiation Time on Turbidity Removal Performance

**Figure 9:**
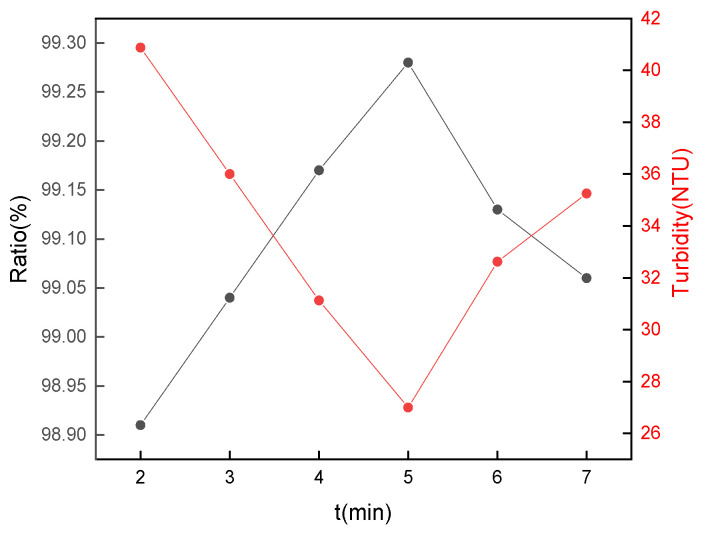
Effect of radiation time on the turbidity removal ratio.

Setting pH = 0.7, microwave power = 80 W, changing the reaction time from 2 min to 7 min with a step length of 1 min. The products are used to treat the simulated wastewater after preparation; the results are displayed in Figure 9.

As can be seen from Figure 9, the turbidity removal ratio of the product gradually increases with the increase of the reaction time from 1 to 5 min, and is highest when the reaction time is 5 min. Because the hydrolysis absorbs heat, the temperature of the system at the beginning of the reaction is low, the degree of hydrolysis reaction is low, and the degree of polymerization of the product is not high at this time. As time increases, the temperature gradually increases [50], the hydrolysis gradually deepens, hydroxide ions gradually replace the chloride ions, promoting the polymerization reaction, while the resulting polymer also has a strong ability to absorb microwaves, which accelerates the polymerization reaction [51], so the degree of polymerization of the product correspondingly increases, and the product of the turbidity performance is also improved. Continue to increase the radiation time, the product of the turbidity ratio gradually decreased, due to the length of time taken to make the polymerization of the product too high or even to generate a high degree of polymerization of hydroxyl complexes, resulting in the product of the turbidity performance decline, so the appropriate time is 5 min.

### 3.8. XRD Analysis of the Products

**Figure 10:**
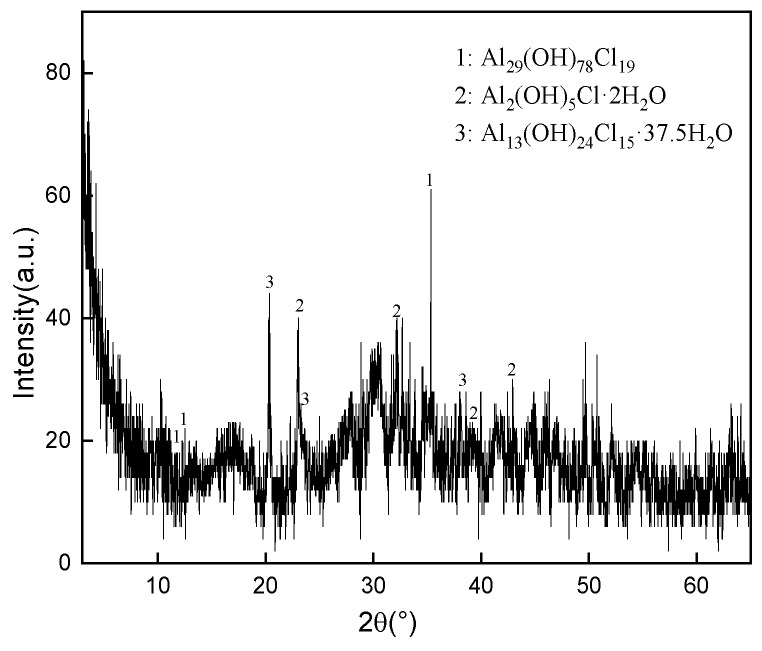
XRD patterns of the product.

The products prepared under the optimized conditions are dried at 50 °C for phase analysis, and the results are shown in Figure 10.

As can be seen from Figure 10, the diffraction peaks of the product are very low in intensity, no strong diffraction peaks of aluminum and iron chloride appear, the main body is diffuse and the spectrum has a high back-bottom, which indicates that the aluminum and iron chloride in the filtrate have polymerized. The diffraction peaks are not high in intensity, indicating that they are predominantly amorphous. Several crystalline polymers of aluminum and iron also appear, e.g., Al_29_(OH)_78_Cl_19_, Al_2_(OH)_5_Cl·2H_2_O, and Al_13_(OH)_24_Cl_15_·37.5H_2_O, but the intensity of the diffraction peaks is low. The difficulty in finding peaks for the iron polymers in Figure 10 suggests that the iron polymers are in an amorphous form, suggesting that the product is a mixture of PAFCs with varying degrees of polymerization. Diffraction peaks of unknown substances also appear in the figure, which is a good indication of the complexity of the composition.

### 3.9. FT-IR Analysis of the Product

**Figure 11:**
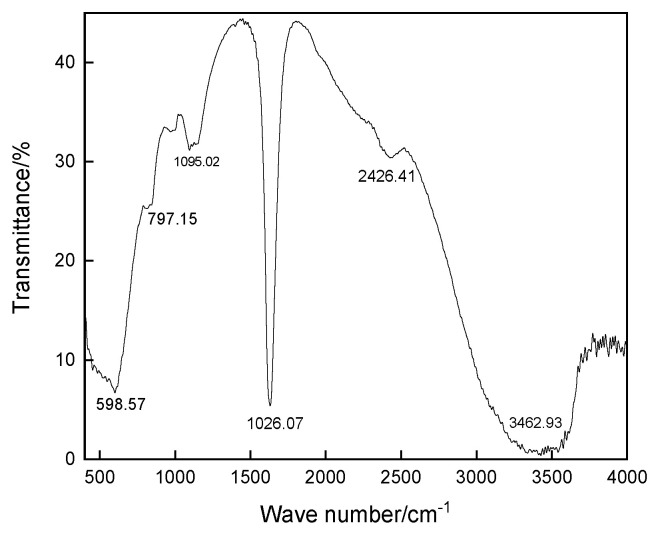
FT-IR spectra of the product.

As can be seen from Figure 11, at 2426.41 cm^−1^ is the Al(Si)-O stretching vibration peak; near 1095.02 cm^−1^ is the Al-O-H-Al stretching vibration peak; at 797.15 cm^−1^ is the Fe-O-H-Fe stretching vibration peak; at 3462.93 cm^−1^ and 1626.07 cm^−1^ are the stretching vibration peaks of H_2_O and -OH The characteristic absorption peak of hydrated chloride is at 598.57 cm^−1^. The analysis of the infrared spectra reveals that PAFC is a hydroxyl-bonded iron-aluminum salt.

### 3.10. Treatment of Mine Wastewater

Referring to the above method, three samples of 800 mL of mine wastewater are taken and tested separately for turbidity removal. The results are shown in Table 2. The turbidity removal performance of PAFC is better than that of PAC and PAF.

## 4. Conclusions

(1) The gangue sample contained 18.78% Al_2_O_3_ and 12.46% Fe_2_O_3_, which belonged to the high iron and low aluminum type gangue.

(2) After calcining the gangue at 675 °C for 1 h, 20% hydrochloric acid is used, 3% calcium fluoride is added and acid leaching occurs at 93 °C for 4 h. The leaching ratio of aluminum ions is 90% and that of iron ions is 91%, and the concentration of aluminum ions in the filtrate is 0.858 mol/L and that of iron ions is 0.366 mol/L.

(3) The optimized process conditions for the preparation of PAFC are: pH = 0.7 of 100 mL acid leach solution is adjusted with calcium carbonate, the power is 80 W, and the time is 5 min. The XRD and FT-IR spectra showed that the prepared product is a mixture of PAFC with different degrees of polymerization.

The preparation of PAFC from coal gangue can realize the resource utilization of gangue. It is mainly used for the treatment of coal industrial wastewater and the treated water can be recycled.

## Figures and Tables

**Table 1:** Main components of the coal gangue, calcined powder, and residue wt %.

Components	Coal Gangue	Calcined Powder	Residue
SiO_2_	44.23	52.1	78
Al_2_O_3_	18.78	21.89	2.84
Fe_2_O_3_	12.46	14.34	1.28
CaO	2.40	2.72	0.02
MgO	0.75	0.84	_
MnO	0.19	0.23	_
P_2_O_5_	0.30	0.34	_
TiO_2_	3.98	4.48	15.5
S	0.31	0.35	_
K_2_O	1.24	1.37	0.78
Na_2_O	0.22	0.24	0.02
FC and others	15.14	1.1	0.92

**Table 2:** Comparison of the effectiveness of PAFC, PAC, and PAF in treating mine water.

	PAC	PAF	PAFC
Turbidity removal ratio/%	96.4	93.7	99.6
residual turbidity/NTU	176.26	308.45	29.38

## Data Availability

The data presented in this study are available on request from the corresponding author.
