# Correction: Kong et al. Preparation of Poly Aluminum-Ferric Chloride (PAFC) Coagulant by Extracting Aluminum and Iron Ions from High Iron Content Coal Gangue. *Materials* 2022, *15*, 2253

**DOI:** 10.3390/ma15093104

**Published:** 2022-04-25

**Authors:** Deshun Kong, Zihan Zhou, Shuojiang Song, Shan Feng, Minglei Lian, Rongli Jiang

**Affiliations:** 1 Guizhou Provincial Key Laboratory of Coal Clean Utilization, School of Chemistry and Materials Engineering, Liupanshui Normal University, Liupanshui 553004, China; lb19040009@cumt.edu.cn (D.K.); lb20040020@cumt.edu.cn (S.S.); 20132013071@cqu.edu.cn (S.F.); feiyuhu2003@126.com (M.L.); 2 School of Chemical Engineering and Technology, China University of Mining and Technology, Xuzhou 221016, China; tb19040017b2@cumt.edu.cn

**Missing Citation**

The authors wish to make the following correction to the published paper [1], the citation mark [13] for “Figure 3. SEM image of the gangue” was lost. The correct Figure 3 caption should be “Figure 3. SEM image of the gangue [13]”.

The authors apologize for any inconvenience caused and state that the scientific conclusions are unaffected. This correction was approved by the Academic Editor. The original publication has also been updated.

## References

[1] Kong D., Zhou Z., Song S., Feng S., Lian M., Jiang R. (2022). Preparation of Poly Aluminum-Ferric Chloride (PAFC) Coagulant by Extracting Aluminum and Iron Ions from High Iron Content Coal Gangue. Materials.

